# Multiple superficial lipomatous nevi coexisting with a verrucous nevus: a case report

**DOI:** 10.3389/fmed.2026.1829263

**Published:** 2026-04-24

**Authors:** Lixiu Chen, Wenjun Zeng, Guihong Zhu, Kun Zhang

**Affiliations:** 1 Department of Dermatology, Dongguan Eastern Central Hospital (The Sixth Affiliated Hospital of Jinan University), Dongguan, China; 2 Department of Otolaryngology, Dongguan Eastern Central Hospital (The Sixth Affiliated Hospital of Jinan University), Dongguan, China

**Keywords:** case report, epidermal nevus, hamartoma, nevus lipomatosus cutaneous superficialis, verrucous nevus

## Abstract

**Background:**

Nevus lipomatosus cutaneous superficialis (NLCS) is a rare benign hamartoma characterized by ectopic mature adipocytes within the dermis. Verrucous epidermal nevus (VEN) is a common epidermal hamartoma presenting with verrucous hyperplasia. The coexistence of these two entities in the same patient is exceedingly rare.

**Case presentation:**

A 20-year-old male presented with an 8-year history of gradually enlarging, asymptomatic papules and nodules on his left lower back. Dermatological examination revealed multiple skin-colored lesions within an area of approximately 24 × 11 cm, arranged in a band-like distribution without crossing the midline. Histopathological examination of an older nodule from the waist demonstrated mature adipose tissue proliferating in lobules within the dermis, consistent with NLCS. Biopsy of a newly developed papule from the back showed epidermal papillomatous hyperplasia, hyperkeratosis, and basal layer hyperpigmentation, consistent with VEN. Based on these findings, the patient was diagnosed with coexistent NLCS and VEN. The larger lesions were surgically excised for cosmetic reasons, and the smaller ones were treated with carbon dioxide laser.

**Conclusion:**

This case highlights the rare coexistence of NLCS and VEN, suggesting a possible common developmental origin, such as a mosaic phenotype affecting both mesenchymal and epidermal lineages. When encountering linear or zosteriform skin lesions, clinicians should consider the possibility of combined hamartomas and perform multiple-site biopsies to avoid underdiagnosis.

## Introduction

Nevus lipomatosus cutaneous superficialis (NLCS) is a rare congenital hamartoma characterized by the aggregation of ectopic mature adipocytes within the dermis (1). It typically presents clinically as clustered, soft, skin-colored or yellowish papules or plaques, often arranged in a zosteriform or linear pattern. The condition was first described by Hoffmann and Zurhelle in 1921 and is therefore also known as Hoffmann–Zurhelle nevus.

Verrucous epidermal nevus (VEN), commonly referred to as epidermal nevus, manifests as linearly arranged verrucous, hyperkeratotic papules or plaques. Histopathologically, it is characterized by epidermal papillomatosis and hyperkeratosis (2).

Although these two entities differ in their tissue of origin (mesenchymal vs. epidermal), both are considered hamartomas resulting from developmental abnormalities. Their coexistence in the same patient is exceedingly rare, with only a few cases reported in the literature. This association may not be coincidental; it suggests that during embryonic development, local pluripotent precursor cells or ectodermal/mesodermal tissues may have simultaneously undergone aberrant signaling, leading to both epidermal hyperplasia and ectopic adipocyte differentiation. Some scholars propose that such lesions fall within the spectrum of a broader category termed “combined cutaneous hamartoma” (3).

## Case report

A 20-year-old male presented to our dermatology department on June 29, 2025, with an 8-year history of gradually enlarging papules and nodules on his left lower back. The lesions initially appeared as rice-grain-sized papules without any identifiable trigger and progressively increased in both number and size over time. Throughout the disease course, the patient reported no associated itching, pain, or ulceration. His medical history was unremarkable, and there was no family history of similar skin conditions or history of trauma or insect bites.

**Figure 1:**
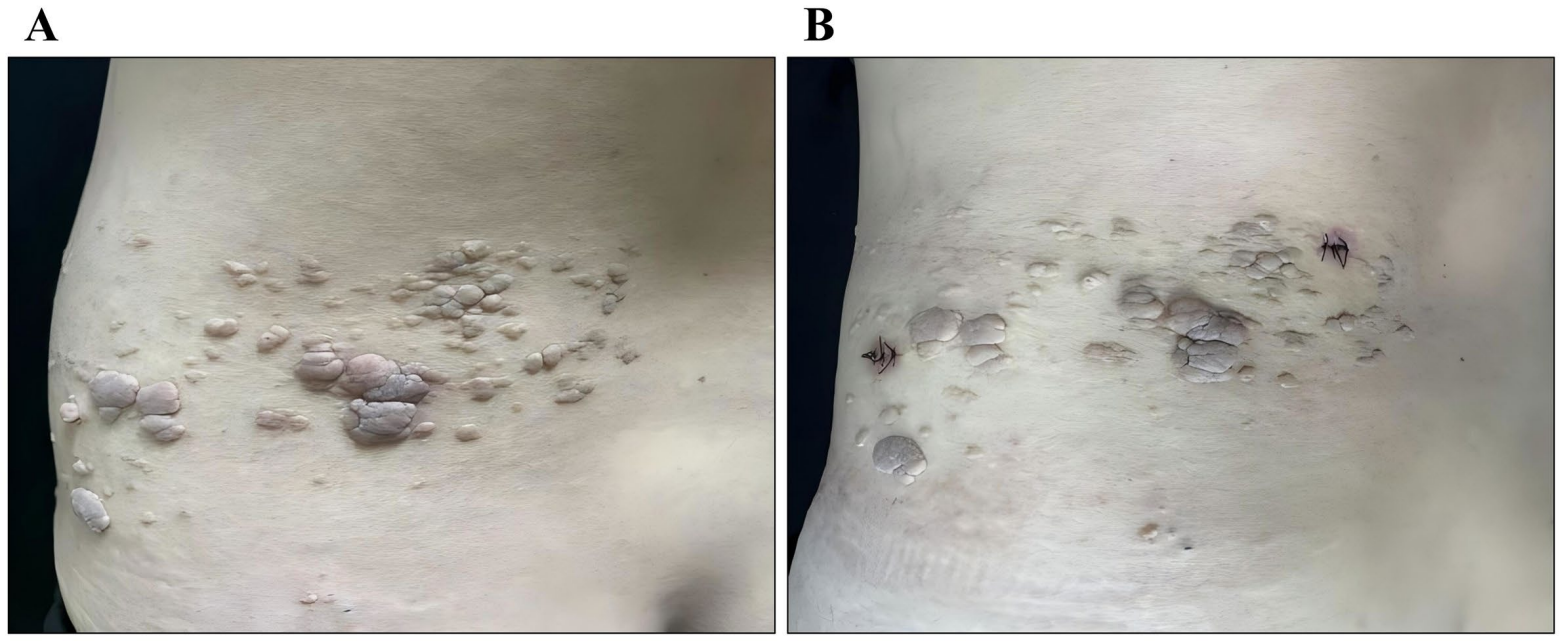
Clinical manifestations of lesions on the left lower back. The lesions cover an area of approximately 24 cm × 11 cm. **(A)** Anterior view (left waist): Multiple skin-colored papules and nodules are visible, scattered in distribution. **(B)** Posterior view (left back): The papules and nodules coalesce into plaques.

Physical examination revealed no significant abnormalities in the cardiopulmonary or abdominal systems. Dermatological examination demonstrated multiple skin-colored papules and nodules confined to an area measuring approximately 24 × 11 cm on the left lumbar and dorsal regions (Figures 1A,B). The lesions ranged in size from 0.3 × 0.2 × 0.2 cm to 2 × 2 × 0.3 cm. They were arranged in a band-like (zosteriform) distribution and did not cross the midline. Some lesions exhibited central depression, while others showed a cerebriform surface. The lesions were slightly soft, well-defined, and non-tender.

**Figure 2:**
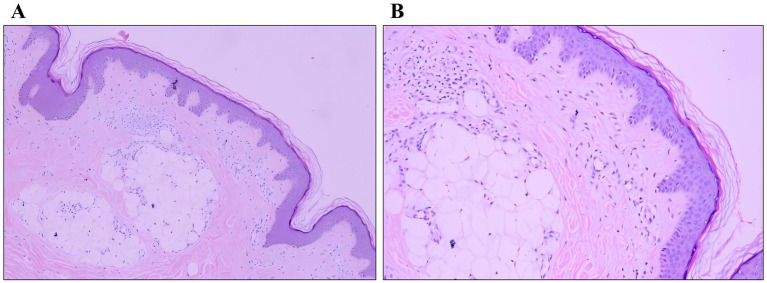
Histopathological findings of the lesion on the left waist. **(A)** Proliferation of fat lobules in the dermis (HE, ×100). **(B)** Collagen fiber hyperplasia and lymphocytic infiltration (HE, ×200).

**Figure 3:**
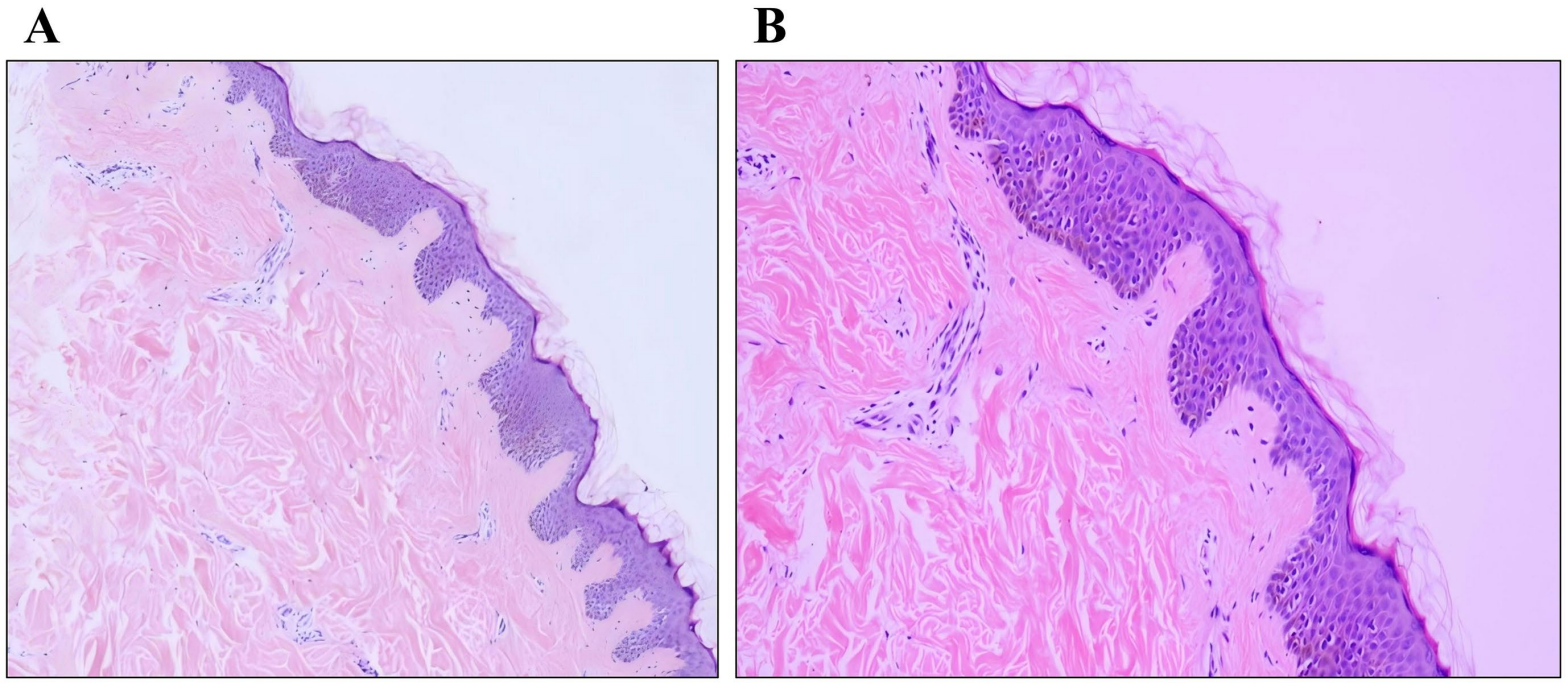
Histopathological findings of the lesion on the back. **(A)** Papillary epidermal hyperplasia (HE, ×100). **(B)** Epidermal hyperkeratosis and basal layer hyperpigmentation (HE, ×200).

Histopathological examination was performed on two representative lesions: an older nodule from the waist and a newly developed papule from the back. The waist lesion showed mature adipose tissue proliferating in lobules within the dermis, consistent with a diagnosis of nevus lipomatosus cutaneous superficialis (NLCS) (Figures 2A,B). The back lesion demonstrated epidermal papillomatous hyperplasia, hyperkeratosis, and focal basal layer hyperpigmentation, accompanied by dermal fibrosis and capillary proliferation, consistent with a diagnosis of verrucous epidermal nevus (VEN) (Figures 3A,B).

For the waist nodule, lipoma (deeper, solitary, no nevus structure), neurofibromatosis (reducible nodules, café-au-lait spots, no fat), and liposarcoma (atypical lipoblasts) were excluded based on clinical and histopathological findings. For the back papule, inflammatory linear verrucous epidermal nevus (prominent inflammation, pruritus), linear porokeratosis (cornoid lamellae), and verruca vulgaris (koilocytes) were ruled out due to the absence of characteristic features. These findings support the diagnoses of NLCS and VEN.

## Discussion

We report a rare case of coexistent nevus lipomatosus cutaneous superficialis (NLCS) and verrucous epidermal nevus (VEN) in a 20-year-old male. The lesions were distributed along Blaschko’s lines on the left lower back, and histopathological examination of two distinct lesions confirmed the diagnosis of both entities. This case is notable not only for the rarity of this association but also for its potential implications regarding the embryonic origin of combined hamartomas.

NLCS is an uncommon benign hamartoma characterized by ectopic mature adipocytes within the dermis. It was first described by Hoffmann and Zurhelle in 1921 and is clinically classified into two subtypes: the classic multiple type and the solitary papular type (4). The classic type typically presents before the age of 30, with clustered or linearly arranged soft papules and plaques preferentially involving the pelvic girdle area, including the buttocks, lumbosacral region, and proximal thighs (5). The solitary type, in contrast, usually occurs in middle-aged or elderly individuals and has no specific predilection site (5). The present case, with onset at age 12 and involvement of the left lumbosacral region, is consistent with the classic type of NLCS.

The pathogenesis of NLCS remains incompletely understood. Several theories have been proposed, including metaplasia of dermal connective tissue into adipose tissue secondary to degenerative changes, heterotopic displacement of adipocytes during embryonic development, and transformation of perivascular mesenchymal cells into mature adipocytes (6). None of these theories, however, fully explain the varied clinical presentations or the occasional association with other hamartomas.

VEN, also known as epidermal nevus, is a common hamartoma of epidermal origin. It typically presents at birth or in early childhood as linear or zosteriform verrucous plaques following Blaschko’s lines (2). Histopathologically, it is characterized by epidermal papillomatosis, hyperkeratosis, and acanthosis, with variable basal layer hyperpigmentation. VEN is believed to result from postzygotic somatic mutations affecting keratinocyte differentiation and proliferation (3).

The coexistence of NLCS and VEN in the same patient is exceptionally rare. While NLCS has been reported in association with various dermal-based lesions—such as dilated pores (7), folliculosebaceous cystic hamartoma (8), hemangioma (9), and nevus sebaceus of Jadassohn (10)—its concurrence with an epidermal hamartoma like VEN has not, to our knowledge, been previously documented. Gutierrez-Gonzalez E et al. (11) described a case of classic NLCS with a verrucous surface, in which histology showed hyperkeratosis, follicular plugging, and mild irregular acanthosis in addition to typical NLCS features. That case demonstrated that epidermal changes can occur secondary to or in association with NLCS. In contrast, the present case exhibited two distinct lesions with independent histopathological features—one purely dermal (NLCS) and one purely epidermal (VEN)-suggesting a true coexistence rather than secondary changes.

The anatomical proximity of the two lesions and their distribution along Blaschko’s lines support the hypothesis of a common mosaic origin. Blaschko’s lines are thought to represent the migration pathways of embryonic ectoderm and mesoderm; thus, lesions following these lines often reflect somatic mosaicism (3). A widely accepted theory proposes that during early embryonic development, a somatic mutation affecting pluripotent precursor cells could lead to the abnormal differentiation of their progeny into two distinct lineages: epidermal (giving rise to VEN) and mesenchymal (giving rise to NLCS). This would result in two different hamartomas arising within the same embryonic segment, a phenomenon referred to as “combined cutaneous hamartoma” (3).

The differential diagnosis of linear or zosteriform lesions on the trunk includes several entities. Linear porokeratosis can present with linearly arranged plaques but is distinguished by the presence of cornoid lamellae on histopathology. Inflammatory linear verrucous epidermal nevus (ILVEN) is characterized by intense pruritus and histopathological evidence of inflammation. Nevus sebaceus of Jadassohn may appear as linear verrucous plaques but typically shows numerous mature sebaceous glands on histology. In the present case, the absence of these features and the distinct histopathological findings in two separate biopsies confirmed the diagnosis of coexistent NLCS and VEN. Recently, a non-invasive diagnostic tool has been proposed for evaluating hamartomatous skin lesions, with a preliminary classification system awaiting validation against histopathology (12). Although histology remains the gold standard, such tools may aid in the clinical differentiation of combined hamartomas.

This case has important clinical implications. First, it underscores the value of multiple-site biopsies when evaluating linear or zosteriform lesions, particularly when lesions show morphological heterogeneity. A single biopsy might have revealed only one of the two hamartomas, leading to an incomplete diagnosis. Second, it highlights the need for clinicians to consider the possibility of combined hamartomas in such presentations, even when the association is exceedingly rare. Finally, this case contributes to the growing body of literature suggesting that some apparently unrelated cutaneous hamartomas may share a common developmental origin.

Regarding management, both NLCS and VEN are benign lesions, and treatment is primarily indicated for cosmetic reasons or to address symptoms such as irritation or maceration. Surgical excision remains the treatment of choice for larger lesions, offering definitive removal with optimal cosmetic outcomes and low recurrence rates (13). For smaller lesions, carbon dioxide laser ablation or topical retinoids may be considered. In the present case, the larger lesions were surgically excised at the patient’s request for cosmetic reasons, and the smaller lesions were treated with carbon dioxide laser, with satisfactory results.

## Conclusion

We report a rare case of coexistent nevus lipomatosus cutaneous superficialis (NLCS) and verrucous epidermal nevus (VEN) in a young adult. The lesions were distributed along Blaschko’s lines on the left lower back, and histopathological examination of two distinct lesions confirmed the diagnosis of both hamartomas. This anatomical proximity and linear distribution suggest a possible common mosaic origin, wherein a somatic mutation affecting pluripotent precursor cells during embryonic development leads to abnormal differentiation into both mesenchymal (NLCS) and epidermal (VEN) lineages within the same segment.

This case highlights two important clinical lessons. First, when evaluating linear or zosteriform skin lesions, clinicians should consider the possibility of combined hamartomas, even when the association is exceedingly rare. Second, multiple-site biopsies are crucial for accurate diagnosis in cases with morphologically heterogeneous lesions, as a single biopsy may reveal only one component and lead to an incomplete diagnosis.

Given the benign nature of both entities, treatment is primarily indicated for cosmetic reasons or symptom relief. Surgical excision remains the preferred option for larger lesions, offering definitive removal with favorable cosmetic outcomes.

## Data Availability

The original contributions presented in the study are included in the article/supplementary material, further inquiries can be directed to the corresponding author.
